# Mirror, mirror on the coast: Exploring body image perception and its nexus with self-esteem, mental well-being among student population at an education hub in South-India

**DOI:** 10.1371/journal.pone.0326171

**Published:** 2025-06-26

**Authors:** Sanjay Kini B, Raghavendra Huchchannavar, Akhila Doddamani, Manjula A, Yash Alok, Sandesh Kini B, Anusha Bhat N, Athulya Krishnan

**Affiliations:** 1 Department of Community Medicine, Kasturba Medical College, Manipal, Manipal Academy of Higher Education, Manipal, Karnataka, India; 2 Department of Community Medicine, KS Hegde Medical Academy, Nitte (Deemed-to-be University), Deralakatte, Mangalore, Karnataka, India; 3 Department of Pediatrics, Kasturba Medical College, Manipal, Manipal Academy of Higher Education, Manipal, Karnataka, India; 4 Srinivas Institute of Medical Sciences and Research Centre, Mukka, Surathkal, Mangalore, Karnataka, India; 5 Prasanna School of Public Health, Manipal, Manipal Academy of Higher Education, Manipal, Karnataka, India; Dilla University College of Health Sciences, ETHIOPIA

## Abstract

**Introduction:**

Young people’s body image dissatisfaction (BID) is a worrying problem that could impact both their physical and mental health. In addition to determining the prevalence and severity of BID among college students in the coastal region of Karnataka, India, this cross-sectional study investigated the relationship between BID and anxiety, depression, and self-esteem.

**Materials and methods:**

Data was gathered using a variety of social media channels from a sample of 382 students who were older than 18 years. The following validated measures were used: the Hamilton Anxiety Rating Scale for anxiety, the Stunkard Figure Rating Scale for depression, the Body Shape Questionnaire-16B for body shape concern, the Rosenberg Self-Esteem Scale for self-esteem and the Patient Health Questionnaire-9 for depression. Additionally, self-reported anthropometric data and sociodemographic information were gathered.

**Results:**

According to the study, 73.03% of participants had a negative perception of their bodies and strongly preferred to have a smaller ideal body image. BID showed a positive correlation with anxiety and depression and a negative correlation with self-esteem. When compared to pupils with normal BMI, those who were overweight or obese showed considerably higher probabilities of being unhappy with their body image. A curious discrepancy between body image dissatisfaction and body form concern was noted, indicating the necessity for a multifaceted evaluation.

**Conclusion:**

The results highlight the significance of providing thorough, evidence-based therapies that are customized to the individual requirements of college students to address body image concerns. Using a comprehensive strategy that incorporates psychological, emotional, and physical health can help students succeed and thrive.

## Introduction

“Mirror, Mirror on the Coast” is a parody of the well-known line from the fairy tale “Snow White,” “Mirror, Mirror on the Wall.” The title links the concept of self-reflection and self-perception (represented by the mirror) with the study’s geographical location (the coastal region of Karnataka) by replacing “the Wall” with “the Coast.” It alludes to an investigation of how young people in this area view their bodies and selves, connecting it to more general issues of body image, self-worth, and mental health among students.

“A person’s perceptions, thoughts, and feelings about his or her body” are referred to as body image. Body image dissatisfaction (BID) is the term used to describe the difference between an individual’s perceived and ideal body image [1]. Individual differences may exist in the degree of this dissatisfaction, and its impact on a person’s life which can range from mild to serious social concerns such as eating disorders, depression, and low self-esteem with widespread negative effects on quality of life [2]. Research on body image has become more and more valuable due to the growing emphasis on physical attractiveness, preoccupation with one’s body image, especially among young people, and the rise in methods to combat dissatisfaction like cosmetic surgery, the use of anabolic steroids, diet pills, Botox, etc [1].

A growing body of research indicates that many young people especially females exhibit greater levels of anxiety about their weight and body image [3]. If these issues are not addressed, they could have a disastrous impact on their adult health in the future. It is believed that body image is a multifaceted notion with behavioural, affective, attitudinal, and perceptual components [4]. The sociocultural context appears to be a significant factor in the emergence of body image distortions and associated pathologies [5]. Many teenagers who are having normal weight feel that they are overweight and obese because they compare themselves to the media’s portrayals of extremely skinny men and women [6]. People who feel discontented about their body image by looking at these pictures may suffer from severe body image dissatisfaction, which is harmful to both their physical and mental health. This discontent may only get worse as the adolescent gets older, peaking in adulthood [7].

Research shows that people who treat themselves with greater compassion and don’t overly link their self-worth to their looks tend to feel more at peace with their bodies [8]. When individuals respond to their struggles and setbacks with understanding and gentleness, they often develop a healthier body image and experience greater emotional wellness [9]. In contrast, some people strongly associate their sense of self-worth to their physical appearance measuring themselves against society’s beauty ideals [10]. For these individuals, their self-esteem can rise and fall based on how they feel about their looks from one moment to another.

Therefore, determining the degree of body image dissatisfaction would be important for the clinical assessment of people who are susceptible to eating disorders, body dysmorphic disorder, substance abuse disorders, anxiety, and depression. The appropriate utilisation of educational programmes to instil positive body image in young people and focus on causes contributing to body image dissatisfaction is another crucial factor to consider. It has been observed that in the region of Coastal Karnataka, India which is considered as students’ hub, there is very little evidence when it comes to college students’ body image and how it relates to their psychological adjustment. Few studies have been conducted earlier among students in this region of coastal Karnataka to assess body image dissatisfaction, but these studies have specifically investigated association of body image dissatisfaction with specific factors like eating disorders or self-worth, and also these studies were conducted mostly among school students [11,12]. There has been no comprehensive study that has evaluated the association of body image dissatisfaction with anxiety, depression, self-esteem, body mass index and also the correlation between all these factors, especially among youth. Hence the present study was conducted to assess the prevalence and magnitude of body image dissatisfaction among college students in coastal Karnataka and to study the association between body image dissatisfaction and self-esteem, anxiety and depression.

## Materials and methods

The present study was a cross-sectional study conducted among the students of Mangalore City in Coastal region of Karnataka State which is considered as students’ Education hub. Students from all over India come for education to Mangalore and it has 7 medical colleges, 20 engineering colleges, 4 law colleges and innumerable colleges offering various other courses. All the students above the age of 18 years studying any disciplines were considered eligible for participation in the study. The study period ranged from October 1^st^ to December 31^st^ 2023. Students were contacted through various social media platforms like Whatsapp, Telegram, Facebook, Twitter etc and were explained the purpose of the study and were administered a pre-tested, semi-structured questionnaire using Google form in English language after obtaining their informed consent, which was provided as an option of “I consent for the study” in google form. Sample size for the study was calculated using the formula for sample size calculation according to prevalence of previous study conducted by Priya D et al [13] in Mangalore among medical students in the year 2007, where the prevalence of body image dissatisfaction was 33.3%. Considering this prevalence, for a precision of 5% and a non-response rate of 10% we obtained a sample size of 381.

The questionnaire used for the study (S1 File) comprised of the following information

i. Socio-demographic information of the study participants- age, gender, marital status, religion, educational qualification, place of residence, type of family, total family income, total no. of persons in the family. Socio-economic status of the study participants was calculated as per the modified BG Prasad classification 2023 [14]. According to this classification per capita income of ₹ 8822 and above is considered as Upper class, ₹ 4411–8821 as upper middle class, ₹ 2647–4410 as middle class, ₹ 1323–2646 as lower middle class and <₹ 1323 as lower class.
ii. Self-reported anthropometric measurements- height (Centimeters), weight (Kgs)
iii. Stunkard figure rating scale which is a psychometric measurement to assess body image satisfaction [15]. The scale presents nine male and nine female schematic silhouettes, ranging from extreme thinness to extreme obesity. The participants were asked to identify the body image they perceive to be having currently and the body image they wish to have. The difference between the two readings represented the body image dissatisfaction. Higher the discrepancy, higher was the body image dissatisfaction.
iv. Body shape questionnaire (BSQ-16 B) which measures an individual’s degree of concern about his/her body shape [16]. The questionnaire consisted of 16 items which were rated on a Likert-type scale. Higher scores pointed to an increased level of concern for body shape. A score of < 80 suggested no concern with shape, 80–110 suggested mild concern, 111–140 suggested moderate concern, and > 140 suggested marked concern.
v. Rosenberg’s Self-Esteem Scale was used to evaluate self-esteem among the study participants, which is the most widely used measure of self-esteem for adult population [17]. The scale composed of 10 items, 5 of which were negatively worded. The scale provided a short, straightforward, and convenient method for measuring global self-esteem. A 4-point Likert scale, spanning from strongly agree to strongly disagree, was used for all item responses. Scores ≤ 20 were categorized as having low self-esteem and those having scores > 20 were categorized as having normal self-esteem.
vi. Hamilton Anxiety rating scale consisted of 14 items, each defined by a series of symptoms, and measured both psychic anxiety (mental agitation and psychological distress) and somatic anxiety (physical complaints related to anxiety) [18]. The total score ranged from 0–56, with a score of 0 (not present) to 4 (severe) for each item. A score of less than 17 denoted light severity, 18–24 mild to moderate severity, and 25–30 severe to moderate.
vii. Depression among the students was assessed using Patient Health Questionnaire-9 (PHQ-9), which scores each of the nine DSM-IV criteria as “0” (not at all) to “3” (nearly every day) [19]. A total score of 1–4 indicated minimal depression, 5–9 indicated mild depression, 10–14 indicated moderate depression, 15–19 indicated moderately severe depression and 20–27 indicated severe depression.

### Statistical analysis

Data was entered using Microsoft excel and analyzed using Statistical Package for Social Sciences (SPSS) version 21. Descriptive statistics were reported using percentages and frequencies. Chi-square test was used to find the association of body image dissatisfaction with socio-demographic variables, BSQ-16 scale, RSE scale, Hamilton anxiety scale, PHQ-9 scale and BMI category. Odd’s ratio with 95% Confidence Interval was calculated to find the association between the various categories of BMI and body image satisfaction. Correlation between various scales namely BSQ-16, RSE, PHQ-9, BMI and body image perception was calculated. The following interpretations were considered for the value of correlation coefficient (r): 1.0 to 0.8: Very strong positive correlation, 0.8 to 0.6: Strong positive correlation, 0.6 to 0.4: Moderate positive correlation, 0.4 to 0.2: Weak positive correlation, 0.2 to 0.0: Very weak positive correlation, 0.0: No correlation, 0.0 to −0.2: Very weak negative correlation, −0.2 to −0.4: Weak negative correlation, −0.4 to −0.6: Moderate negative correlation, −0.6 to −0.8: Strong negative correlation, −0.8 to −1.0: Very strong negative correlation.

### Ethical clearance

The Institutional Ethics Committee (IEC) of K.S. Hegde Medical Academy, Mangalore, India, reviewed and approved the study protocol via letter no: IEC. Citation: INST.EC/EC/223/2023, dated September 15, 2023. The study was conducted in accordance with the ethical guidelines outlined in the Declaration of Helsinki, the international ethical framework for research involving human subjects.

## Results

Among the study participants 52.9% were females and 47.1% were males. Majority of the students (95.8%) were unmarried and belonged to Hindu religion (88.5%), were perusing graduation (86.9%), belonged to nuclear family (86.1%) and belonged to upper socio-economic status (84.6%). The socio-demographic information of the study participants is depicted in Table 1.

**Table 1:** Socio-demographic variables of study participants (N = 382).

Socio-demographic variables	Frequency	Percentage
Gender.		
Female	202	52.9
Male	180	47.1
Marital Status:		
Married	10	2.6
Unmarried	366	95.8
Prefer not to disclose	6	1.6
Religion:		
Hindu	338	88.5
Muslim	19	5.0
Christian	24	6.3
Others	1	0.3
Educational Qualification:		
Graduation	332	86.9
Post-graduation and above	50	13.1
Type of family:		
Nuclear	329	86.1
Joint	38	9.9
3 Generation	15	3.9
Socio-Economic Status:		
Upper class	323	84.6
Upper middle class	41	10.7
Lower middle class	13	3.4
Lower class	5	1.3

The present study depicted that 279 (73.03%) participants were dissatisfied with their body image. It was observed that students desiring to have a “smaller (thinner)” ideal image as compared to their perceived image (real image as they perceive themselves as) was higher both among married as well as unmarried participants. Among the unmarried students 54.4% of students desired to have a smaller ideal image compared to their perceived image whereas 18.3% desired to have larger ideal image compared to perceived image. Among the students who were married 80% of the participants desired a smaller ideal image compared to only 10% of the participants who desired a larger ideal image. This result was statistically significant (p < 0.001). However, when we looked association of sociodemographic variables with the overall proportion of participants who were satisfied with their body image and those who were dissatisfied, the results were not statistically significant. Table 2 represents the association between Socio-demographic variables and Body image dissatisfaction.

**Table 2:** Association between Socio-demographic variables and Body image dissatisfaction (N = 382).

Socio-demographic variables		Body image Dissatisfaction [Frequency (%)]									P value	Satisfied	Dissatisfied	P value
		−3.00	−2.00	−1.00	0.00	1.00	2.00	3.00	4.00	Total				
Gender	Female	2 (1)	7 (3.5)	27 (13.4)	52 (25.7)	68 (33.7)	37 (18.3)	9 (4.5)	0 (0.0)	202 (100.0)	0.809	52 (25.7)	150 (74.3)	0.644
	Male	1 (0.6)	6 (3.3)	26 (14.4)	51 (28.3)	51 (28.3)	34 (18.9)	9 (5.0)	2 (1.1)	180 (100.0)		51 (28.3)	129 (71.7)	
Marital status	Married	1 (10.0)	0 (0.0)	0 (0.0)	1 (10.0)	2 (20.0)	3 (30.0)	2 (20.0)	1 (10.0)	10 (100.0)	**<0.001**	1 (10.0)	9 (90.0)	0.544
	Prefer not to disclose	0 (0.0)	0 (0.0)	1 (16.7)	2 (33.3)	1 (16.7)	0 (0.0)	2 (33.3)	0 (0.0)	6 (100.0)		2 (33.3)	4 (66.7)	
	Unmarried	2 (0.5)	13 (3.6)	52 (14.2)	100 (27.3)	116 (31.7)	68 (18.6)	14 (3.8)	1 (0.3)	366 (100.0)		100 (27.3)	266 (72.7)	
Educational qualification	Graduation	2 (0.6)	12 (3.6)	45 (13.6)	86 (25.9)	107 (32.2)	61 (18.4)	17 (5.1)	2 (0.6)	332 (100.0)	0.677	86 (25.9)	246 (74.1)	0.235
	Post-graduation and above	1 (2.0)	1 (2.0)	8 (16.0)	17 (34.0)	12 (24.0)	10 (20.0)	1 (2.0)	0 (0.0)	50 (100.0)		17 (34.0)	33 (66.0)	
Type of family	Three-generation	0 (0.0)	1 (6.7)	2 (13.3)	4 (26.7)	3 (20.0)	4 (26.7)	1 (6.7)	0 (0.0)	15 (100.0)	0.798	4 (26.7)	11 (73.3)	1.000
	Joint	0 (0.0)	3 (7.9)	4 (10.5)	10 (26.3)	13 (34.2)	6 (15.8)	1 (2.6)	1 (2.6)	38 (100.0)		10 (26.3)	28 (73.7)	
	Nuclear	3 (0.9)	9 (2.7)	47 (14.3)	89 (27.1)	103 (31.3)	61 (18.5)	16 (4.9)	1 (0.3)	329 (100.0)		89 (27.1)	240 (72.9)	
Socio-economic status	Upper class	3 (0.9)	10 (3.1)	43 (13.3)	89 (27.6)	95 (29.4)	65 (20.1)	16 (5.0)	2 (0.6)	323 (100.0)	0.827	89 (27.6)	234 (72.4)	0.779
	Upper middle class	0 (0.0)	2 (4.9)	9 (22.0)	9 (22.0)	16 (39.0)	4 (9.8)	1 (2.4)	0 (0.0)	41 (100.0)		9 (22.0)	32 (78.0)	
	Lower middle class	0 (0.0)	1 (7.7)	1 (7.7)	3 (23.1)	6 (46.2)	2 (15.4)	0 (0.0)	0 (0.0)	13 (100.0)		3 (23.1)	10 (76.9)	
	Lower class	0 (0.0)	0 (0.0)	0 (0.0)	2 (40.0)	2 (40.0)	0 (0.0)	1 (20.0)	0 (0.0)	5 (100.0)		2 (40.0)	3 (60.0)	

When the students were administered the BSQ-16 questionnaire to assess the degree of concern about their body shape, it was observed that even among the students who had no concern 54.5% of participants desired to have a smaller ideal image compared to their perceived image whereas only 18.1% of participants desired to have a larger ideal image compared to their perceived image. Among the students who had mild concern about their body image, 83.3% of the participants desired to have a smaller ideal image whereas only 3.3% of participants desired to have a larger ideal image and this result was statistically significant (p = 0.017).

Results from the PHQ-9 scale to assess the amount of depression in students depicted that among those who had moderate to severe depression three-fourth of the students desired to have a smaller ideal image compared to their perceived image whereas none of them desired a larger ideal image compared to their perceived image. Among those participants who had moderate depression it was observed that 62.9% desired to have a smaller ideal image compared to their perceived image, whereas 11.1% of participants desired to have a larger ideal image compared to their perceived image. Among the participants with mild depression, 60.3% desired to have a smaller ideal image compared to their perceived image, whereas only 16.1% of students desired to have a larger ideal image compared to their perceived image. 51.5% of the participants in the category of minimal depression desired to have a smaller ideal image compared to their perceived image, whereas only 22.7% desired to have a larger ideal image compared to their perceived image. Even among those participants who had no depression, it was observed that 53.0% desired to have a smaller ideal image compared to their perceived image whereas only 17.2% of the participants desired to have a larger ideal image compared to their perceived image and this result was statistically significant (p = 0.014). However, when we looked at the association of the results from various scales like BSQ-16, RSE scale, Hamilton anxiety scale and PHQ-9 scale with the overall proportion of participants who were satisfied and dissatisfied with their body image, the results were not statistically significant.

With respect to the BMI category of the study participants, it was observed that among the obese students 90% of the students desired to have a smaller ideal image as compared to their perceived image, whereas none of them desired to have a larger ideal image as compared to their perceived image. Among those who were overweight 84.0% of the students desired to have a smaller ideal image as compared to their perceived image, whereas 2.8% of students desired to have a larger ideal image as compared to their perceived image. 51.7% of the participants in the category of normal BMI also desired to have a smaller ideal image as compared to their perceived image, whereas 14.7% of students desired to have a larger ideal image as compared to their perceived image. But among those students who were underweight 55.3% of students desired to have a larger ideal image as compared to their perceived image whereas only 20.6% of the participants desired to have a smaller ideal image as compared to their perceived image. The aforementioned results were statistically significant (p < 0.001). Similarly, when we looked at the proportion of students in each category of BMI, it was observed that a higher proportion of students were dissatisfied with their body image compared to those who were satisfied, and the results were statistically significant (p = 0.01). The association of various scales vs body image dissatisfaction is mentioned in Table 3.

**Table 3:** Association of various scales vs Body image dissatisfaction (N = 382).

Scales/Category		Body image Dissatisfaction [Frequency (%)]									P value	Satisfied	Dissatisfied	P value
		−3.00	−2.00	−1.00	0.00	1.00	2.00	3.00	4.00	Total				
BSQ-16	No concern	3 (0.8)	13 (3.5)	53 (14.1)	102 (27.1)	119 (31.6)	68 (18.1)	16 (4.3)	2 (0.5)	376 (100.0)	**0.017**	102 (27.1)	274 (72.9)	1.000
	Mild concern	0 (0.0)	0 (0.0) (3.3)	0 (0.0)	1 (16.7)	0 (0.0)	3 (50.0)	2 (33.3)	0 (0.0)	6 (100.0)		1 (16.7)	5 (83.3)	
RSE Scale	Low self esteem	0 (0.0)	6 (6.7)	13 (14.6)	17 (19.1)	24 (27.0)	22 (24.7)	6 (6.7)	1 (1.1)	89 (100.0)	0.093	17 (19.1)	72 (80.9)	0.058
	Normal self esteem	3 (1.0)	7 (2.4)	40 (13.7)	86 (29.4)	95 (32.4)	49 (16.7)	12 (4.1)	1 (0.3)	293 (100.0)		86 (29.4)	207 (70.6)	
Hamilton Anxiety Scale	Mild Anxiety	3 (1.1)	10 (3.8)	39 (14.8)	75 (28.4)	86 (32.6)	40 (15.2)	9 (3.4)	2 (0.8)	264 (100.0)	0.228	75 (28.4)	189 (71.6)	0.643
	Moderate Anxiety	0 (0.0)	2 (3.5)	4 (7.0)	13 (22.8)	19 (33.3)	16 (28.1)	3 (5.3)	0 (0.0)	57 (100.0)		13 (22.8)	44 (77.2)	
	Severe Anxiety	0 (0.0)	1 (1.6)	10 (16.4)	15 (24.6)	14 (23.0)	15 (24.6)	6 (9.8)	0 (0.0)	61 (100.0)		15 (24.6)	46 (75.4)	
PHQ9 scale	No depression	2 (1.3)	5 (3.3)	19 (12.6)	45 (29.8)	56 (37.1)	17 (11.3)	5 (3.3)	2 (1.3)	151 (100.0)	**0.014**	45 (29.8)	106 (70.2)	0.896
	Minimal depression	1 (0.8)	5 (3.9)	23 (18.0)	33 (25.8)	38 (29.7)	25 (19.5)	3 (2.3)	0 (0.0)	128 (100.0)		33 (25.8)	95 (74.2)	
	Mild depression	0 (0.0)	2 (2.9)	9 (13.2)	16 (23.5)	18 (26.5)	19 (27.9)	4 (5.9)	0 (0.0)	68 (100.0)		16 (23.5)	52 (76.5)	
	Moderate depression	0 (0.0)	1 (3.7)	2 (7.4)	7 (25.9)	5 (18.5)	9 (33.3)	3 (11.1)	0 (0.0)	27 (100.0)		7 (25.9)	20 (74.1)	
	Moderately severe depression	0 (0.0)	0 (0.0)	0 (0.0)	2 (25.0)	2 (25.0)	1 (12.5)	3 (37.5)	0 (0.0)	8 (100.0)		2 (25.0)	6 (75.0)	
BMI Category	Underweight	1 (1.7)	8 (13.8)	23 (39.7)	14 (24.1)	9 (15.5)	2 (3.4)	1 (1.7)	0 (0.0)	58 (100.0)	**<0.001**	14 (24.1)	44 (75.9)	**0.01**
	Normal	1 (0.4)	5 (2.2)	28 (12.1)	78 (33.6)	80 (34.5)	33 (14.2)	7 (3.0)	0 (0.0)	232 (100.0)		78 (33.6)	154 (66.4)	
	Overweight	1 (1.4)	0 (0.0)	1 (1.4)	9 (13.0)	22 (31.9)	26 (37.7)	9 (13.0)	1 (1.4)	69 (100.0)		9 (13.0)	60 (87.0)	
	Obese	0 (0.0)	0 (0.0)	0 (0.0)	2 (10.0)	7 (35.0)	9 (45.0)	1 (5.0)	1 (5.0)	20 (100.0)		2 (10.0)	18 (90.0)	

In the present study it was observed that the odds of students being dissatisfied with their body image was 3.29 times more associated with the overweight category of participants when compared to the students with normal BMI. Table 4 depicts the association between BMI and body image.

**Table 4:** Association between BMI and body image.

Grades of BMI	Satisfied	Dissatisfied	Odd’s ratio	95% CI
Normal	78 (33.6)	154 (66.4)	1	**–**
Underweight	14 (24.1)	44 (75.9)	1.55	(0.80- 3.00)
Overweight	9 (13.0)	60 (87.0)	3.29	(1.55-6.98)
Obese	2 (10.0)	18 (90.0)	2.96	(0.84-10.36)

There was negative correlation between BMI and RSE scores, BSQ-16B and RSE scores, RSE scores with body image perception and RSE with PHQ-9 scores. The correlation between various scales used in the study has been reported in Table 5.

**Table 5:** Correlation between various scales (N = 382).

		Body image perception	BMI	BSQ-16B	RSE score	PHQ-9 score
Body image perception	Correlation coefficient	1.000	0.533	0.573	0.121	0.139
	p value	–	0.000	0.000	0.018	0.007
BMI	Correlation coefficient	0.533	1.000	0.387	−0.083	0.138
	p value	0.000	–	0.000	0.105	0.007
BSQ-16B	Correlation coefficient	0.573	0.387	1.000	−0.351	0.347
	p value	0.000	0.000	–	0.000	0.000
RSE score	Correlation coefficient	−0.121	−0.083	−0.351	1.000	−0.365
	p value	0.018	0.105	0.000	–	0.000
PHQ-9 score	Correlation coefficient	0.139	0.138	0.347	−0.365	1.000
	p value	0.007	0.007	0.000	0.000	–

## Discussion

The purpose of the current study was to evaluate the frequency and severity of body image dissatisfaction among college students in coastal Karnataka and its relationship to anxiety, depression, and self-esteem. It is alarming to note that three-fourths of the students in our study were unhappy with their bodies, especially with their perceived existing bodies and with their wish to be slimmer. We had almost an equal proportion of male and female students in our study. In the present study it was found that, for both married and single individuals, students’ desire for a “smaller (thinner)” ideal image relative to their perceived image was higher, which is in concurrence with the study conducted by Cunha CM et al [20] in Brazil which reported that people who were married or in a stable union had a higher prevalence of body image dissatisfaction. Even though we did not find any association between gender and body image dissatisfaction, the findings of a systematic review and meta-analysis showed that there was a substantial correlation between female gender and body image dissatisfaction [2]. The reason for the craving to be thin may be evident from studies such as the ones conducted by Jiosta B et al [21] in France, which found that young women, who are particularly susceptible to the ideals of thinness, often associate thinness with success and beauty. A study conducted by Latiff A et al [22] among primary school-going children of an orthodox Malay community also showed that 60.1% of the students had body image dissatisfaction and that female students reported a higher body image dissatisfaction (66.1%) compared to males. Surprisingly in contrast to most of the studies, research conducted by Alharballeh S et al [23] among youth in United Arab Emirates showed that men reported a higher prevalence of body image dissatisfaction than females which may be because in the traditional Arab communities plump women are considered more attractive which minimizes their pressure to lose weight and look thinner.

Socioeconomic status did not have any bearing on the proportion of body image dissatisfaction in the current study. The results are consistent with the earlier studies which state that Body image dissatisfaction is not associated with any particular strata of socio-economic groups and is universally found in all [24–26]. Educational qualification of the students had no role to play with regards to body image dissatisfaction in our study but a few studies have varying results which reported that people with higher educational qualifications (Professors) had a higher proportion of body image dissatisfaction than undergraduate students [20]. Six students (1.5%) in the current study expressed minor concerns about their body shape when we employed the BSQ-16 questionnaire to gauge students’ concerns about body shape. In contrast to our research, AL-Otaibi H et al.’s [27] study of Saudi Arabian university students revealed that, when the BSQ scale was used, 16.8% of the students reported being mildly concerned about their body shape, 8.9% reported being moderately worried, and 2.4% reported being extremely worried. One interesting observation made in our study is the fact that although majority of the students expressed no concern with their body shape, but significant proportion of students were dissatisfied with their body image. The possible explanation for these findings can be attributed to the fact that the BSQ-16 may not be as comprehensive or sensitive to subtleties as the Stunkard figure rating scale when it comes to body image dissatisfaction. Responses to body image and shape may be influenced by cultural norms and standards. Some of the participants in our study probably feel unsatisfied with their overall body image because of other criteria like weight, attractiveness, or comparison to societal ideals, even though they may not openly express concern about their body shape because of societal expectations or norms.

The current study also highlights how students’ self-esteem declines as the proportion of students who are dissatisfied with their bodies increases. This is demonstrated by the negative correlation between Rosenberg’s self-esteem scale and other measures like body image perception and the BSQ-16B scale. Several studies conducted in India [28,29] and abroad [30,31] concerning self-esteem and body image dissatisfaction have seconded our results and in light of these findings, it is likely that students who exhibit both high levels of self-consciousness and low self-esteem will be more likely to criticize themselves and, as a result, be less satisfied with their bodies. Self-esteem encourages people to be resilient during difficult times and plays a supporting role in overcoming life’s obstacles. People who are confident in themselves show optimism and resist giving in to unreasonable social norms. It is important to note that success does not always equate to having a flawless appearance in the eyes of self-assured individuals. Our study did not find any significant association between body image dissatisfaction and anxiety as measured by Hamilton anxiety scale. A research study conducted by Diengdoh I et al [32] among college students in northeastern India, reported that anxiety and low self-esteem were major predictors of body image dissatisfaction.

Although in the present study, we did not find any significant association between body image dissatisfaction and depression (using PHQ-9 scale), it was observed that among all the categories of depression whether mild or severe, there was a desire to have slimmer ideal weight and this trend was statistically significant (p = 0.014). Flores-Cornejo F et al [33] in their study among adolescents in Lima, Peru found a statistically significant association between body image dissatisfaction and depression (using PHQ-9 scale). This is in line with earlier research that discovered a correlation between these characteristics, including studies conducted among Turkish teenagers by Ozmen D et al [34] in 2007 and Almeida S et al [35] in Portugal in 2013 which means that even a decade ago the trends remained the same. Results of our study also proved the fact that BMI was a significant correlate of Body image dissatisfaction. There was a dose-response relationship observed between various categories of BMI and body image dissatisfaction. It is fascinating to note that the odd’s of body image dissatisfaction were 1.55 times higher among underweight individuals compared to those having normal BMI, whereas it was 3.29 times higher among overweight and 2.96 times higher among obese individuals, which means that students are more dissatisfied with their body image if they are overweight or obese than when they are underweight. This is one unique comparison that we have observed in our study which is not found in the research studies conducted earlier in the literature. A comprehensive review and meta-analysis using 17 publications was conducted by Weinberger NA et al [2] to methodically compare the level of body image dissatisfaction in obese people to that in people of normal weight and examine gender differences in body image dissatisfaction. Compared to the group with normal weight, the obese group saw a considerable increase in body image dissatisfaction, as demonstrated by this meta-analysis. It also showed that women with obesity and those with a normal weight have considerably different levels of body image dissatisfaction than men. Additionally, our findings concur with research conducted worldwide. Divecha CA et al [36] in Oman found a strong correlation between self-reported BMI and perceived BMI as well as body image dissatisfaction among medical students. The results are also comparable to the study by Latiff et al [17] in Malaysia that found students who were overweight or obese had a four times higher risk of developing BID compared to students who were having normal weight or were underweight. Interestingly a study conducted by Mohammed BAA et al [37] among female medical students in Sudan found that underweight students had more body image dissatisfaction compared to their overweight counterparts. The study revealed that 55% of respondents consistently expressed sensitivity about others perceiving them as thin, while 73% self-identified themselves as thin and desired increased body mass in specific areas. The social norms in Africa, which view obesity as a sign of prosperity and contentment, may have an impact on this study result. Despite modernity and Westernization, this notion is still deeply ingrained in African societies.

To evaluate the important covariates of body image dissatisfaction, our study used validated and well-established instruments such as the Hamilton Anxiety Rating Scale, Body Shape Questionnaire (BSQ-16B), Stunkard Figure Rating Scale, and Rosenberg Self-Esteem Scale which highlights the strength and robustness of the research study. The study provides a comprehensive picture of the problem by examining the complex relationships between body image dissatisfaction and several variables, including self-esteem, academic performance, anxiety, and depression. The research strengthens the evidence by demonstrating a dose-response relationship between body image dissatisfaction and various BMI categories. This work is highly relevant and tackles a pressing issue in coastal Karnataka, an education hub catering to thousands of students from across India and overseas and notably at a stage where a medical student recently committed suicide due to her self-inflicted frustration with her appearance and increase in body weight [38]. Some of the limitations of the present study are – The Stunkard figure rating scale, which may not adequately reflect the multifaceted nature of this construct, was the main tool employed in the study to measure body image dissatisfaction. To clarify the lived experiences and contextual elements influencing body image views, the study exclusively used quantitative methodologies, which prevented it from offering the depth and nuanced insight that qualitative approaches may have, which paves the way for future research in this area.

## Conclusion

According to the study, a sizable fraction of college students in the coastal Karnataka region (73.33%) were dissatisfied with their bodies and strongly preferred their ideal, slimmer bodies to their actual, observed body shapes. The high rate of body image dissatisfaction among college students, especially those who are overweight or obese, highlights the need for focused interventions and educational initiatives to support healthy lifestyle choices, a positive body image, and increased self-esteem. College students’ unhappiness with their bodies necessitates a multidisciplinary response from educators, counsellors, doctors, and legislators. This cooperative effort can support the creation and application of successful intervention techniques. The main goals of interventions are to advance self-acceptance, body positivity, and a broader, inclusive concept of beauty that defies conventional norms. Peer support initiatives, media literacy programmes, and awareness campaigns can all be part of this. Interventions should provide children with appropriate coping strategies to address issues related to body image, such as resilience development, stress management, and cultivating a strong sense of self-worth that is not based on looks.

## Supporting information

S1 File Perception of body image and its association with self-esteem, anxiety and depression among students. (DOCX)

## References

[1] Grogan S. Body image: understanding body dissatisfaction in men, women and children. 3rd ed. Routledge; 2016. doi: 10.4324/9781315681528

[2] Weinberger N-A, Kersting A, Riedel-Heller SG, Luck-Sikorski C. Body dissatisfaction in individuals with obesity compared to normal-weight individuals: a systematic review and meta-analysis. Obes Facts. 2016;9(6):424–41. doi: 10.1159/000454837 28013298 PMC5644896

[3] Heron KE, Smyth JM, Akano E, Wonderlich SA. Assessing body image in young children. Sage Open. 2013;3(1):215824401347801. doi: 10.1177/2158244013478013

[4] Body image: perceptions, interpretations and attitudes [Internet]. New York: Nova Science Publishers; 2011 [cited 2025 Apr 30]. Available from: https://books.google.co.in/books/about/Body_Image.html?id=wPoekgAACAAJ&redir_esc=y

[5] Hosseini SA, Padhy RK. Body image distortion (archived). 2023 Sep 4. In: StatPearls [Internet]. Treasure Island (FL): StatPearls Publishing; 2025 [cited 2025 Apr 30]. 31536191

[6] Chang F-C, Lee C-M, Chen P-H, Chiu C-H, Pan Y-C, Huang T-F. Association of thin-ideal media exposure, body dissatisfaction and disordered eating behaviors among adolescents in Taiwan. Eat Behav. 2013;14(3):382–5. doi: 10.1016/j.eatbeh.2013.05.002 23910785

[7] Bucchianeri MM, Arikian AJ, Hannan PJ, Eisenberg ME, Neumark-Sztainer D. Body dissatisfaction from adolescence to young adulthood: findings from a 10-year longitudinal study. Body Image. 2013;10(1):1–7. doi: 10.1016/j.bodyim.2012.09.001 23084464 PMC3814026

[8] Homan KJ, Tylka TL. Self-compassion moderates body comparison and appearance self-worth’s inverse relationships with body appreciation. Body Image. 2015;15:1–7. doi: 10.1016/j.bodyim.2015.04.007 25978272

[9] Neff KD. The development and validation of a scale to measure self-compassion. Self Identity. 2003;2(3):223–50. doi: 10.1080/15298860309027

[10] Stapleton P, Crighton GJ, Carter B, Pidgeon A. Self-esteem and body image in females: the mediating role of self-compassion and appearance contingent self-worth. Humanist Psychol. 2017;45(3):238–57. doi: 10.1037/hum0000059

[11] Thomas C, D’Souza A, Priyadarshini S. Determining the perceived body image and dieting attitude among high school students in selected schools of Udupi district. IJPHRD. 2015;6(1):137. doi: 10.5958/0976-5506.2015.00027.3

[12] KS L, Hegde S, Sharma P, Rai P. Body image, self-esteem and depression in female adolescent college students. J Indian Assoc Child Adolesc Ment Health. 2006;2(3):78–84. doi: 10.1177/0973134220060303

[13] Priya D, Prasanna KS, Sucharitha S, Vaz NC. Body image perception and attempts to change weight among female medical students at Mangalore. Indian J Community Med. 2010;35(2):316–20. doi: 10.4103/0970-0218.66886 20922115 PMC2940194

[14] Ghodke M. Updated BG Prasad’s socioeconomic status classification for the year 2023. Indian J Community Med. 2023;48(6):934–6. doi: 10.4103/ijcm.ijcm_401_23 38249702 PMC10795881

[15] Scagliusi FB, Alvarenga M, Polacow VO, Cordás TA, de Oliveira Queiroz GK, Coelho D, et al. Concurrent and discriminant validity of the Stunkard’s figure rating scale adapted into Portuguese. Appetite. 2006;47(1):77–82. doi: 10.1016/j.appet.2006.02.010 16750589

[16] Evans C, Dolan B. Body shape questionnaire: derivation of shortened “alternate forms”. Int J Eat Disord. 1993;13(3):315–21. doi: 10.1002/1098-108x(199304)13:3<315::aid-eat2260130310>3.0.co;2-3 8477304

[17] Martín-Albo J, Núñiez JL, Navarro JG, Grijalvo F. The Rosenberg Self-Esteem Scale: translation and validation in university students. Span J Psychol. 2007;10(2):458–67. doi: 10.1017/s1138741600006727 17992972

[18] Shear MK, Vander Bilt J, Rucci P, Endicott J, Lydiard B, Otto MW, et al. Reliability and validity of a structured interview guide for the Hamilton Anxiety Rating Scale (SIGH-A). Depress Anxiety. 2001;13(4):166–78. 11413563

[19] Kroenke K, Spitzer RL, Williams JB. The PHQ-9: validity of a brief depression severity measure. J Gen Intern Med. 2001;16(9):606–13. doi: 10.1046/j.1525-1497.2001.016009606.x 11556941 PMC1495268

[20] Cunha C de M, Pereira EM, Souto MCR, de Sá LB, da Silva HBM, de Brito E, et al. Factors associated with body image dissatisfaction in a Brazilian university sample during the COVID-19 pandemic. Front Educ. 2023;8:1044727. doi: 10.3389/feduc.2023.1044727

[21] Jiotsa B, Naccache B, Duval M, Rocher B, Grall-Bronnec M. Social media use and body image disorders: association between frequency of comparing one’s own physical appearance to that of people being followed on social media and body dissatisfaction and drive for thinness. Int J Environ Res Public Health. 2021;18(6):2880. doi: 10.3390/ijerph18062880 33799804 PMC8001450

[22] Latiff AA, Muhamad J, Rahman RA. Body image dissatisfaction and its determinants among young primary-school adolescents. J Taibah Univ Med Sci. 2017;13(1):34–41. doi: 10.1016/j.jtumed.2017.07.003 31435300 PMC6694944

[23] Alharballeh S, Dodeen H. Prevalence of body image dissatisfaction among youth in the United Arab Emirates: gender, age, and body mass index differences. Curr Psychol. 2023;42(2):1317–26. doi: 10.1007/s12144-021-01551-8 33679115 PMC7919234

[24] Deleel ML, Hughes TL, Miller JA, Hipwell A, Theodore LA. Prevalence of eating disturbance and body image dissatisfaction in young girls: an examination of the variance across racial and socioeconomic groups. Psychol Sch. 2009;46(8):767–75. doi: 10.1002/pits.20415 20336184 PMC2844708

[25] Regnier F, Le Bihan E, Tichit C, Baumann M. Adolescent body dissatisfaction in contrasting socioeconomic milieus, coming from a french and luxembourgish context. Int J Environ Res Public Health. 2019;17(1):61. doi: 10.3390/ijerph17010061 31861763 PMC6982110

[26] Liyanage G, Karunainathan T, Jeyarajah L, Thevatheepan P, Thavendra M, Seneviwickrama M. Body image dissatisfaction and its determinants in urban Sri Lankan adolescents. Ceylon Med J. 2021;66(4):185–90. doi: 10.4038/cmj.v66i4.9509 35570350

[27] AL-Otaibi HH, Nassef SL, Raouf TA. Body shape dissatisfaction, weight status and physical activity among a sample university students in Saudi Arabia. FNS. 2013;04(06):616–25. doi: 10.4236/fns.2013.46079

[28] Paria B, Dwivedi M, Roy SK. A cross-sectional study on body image perception and self-esteem among adolescent girls in urban and rural areas of Kolkata, West Bengal, India. JCDR. 2023. doi: 10.7860/jcdr/2023/62667.18697

[29] Sampath H, Soohinda G, Mishra D, Dutta S. Body image dissatisfaction in young Indian Men: prevalence, psychosocial correlates, and the impact of sociocultural pressure. Indian J Soc Psychiatry. 2020;36(2):130. doi: 10.4103/ijsp.ijsp_28_19

[30] Jakobek V, Kranjčev M, Barić R. Predictors of body image dissatisfaction in kinesiology students. Front Psychol. 2024;14:1322553. doi: 10.3389/fpsyg.2023.1322553 38379844 PMC10878395

[31] Shahyad S, Pakdaman S, Shokri O. Prediction of body image dissatisfaction from self-esteem, thin-ideal internalization and appearance-related social comparison. IJTMGH. 2015;3(2):59–63. doi: 10.20286/ijtmgh-030299

[32] Diengdoh I, Ali A. Body image and its association with depression, anxiety, and self-esteem among college going students: a study from Northeast India. Indian J Community Med. 2022;47(2):218–22. doi: 10.4103/ijcm.ijcm_881_21 36034238 PMC9400361

[33] Flores-Cornejo F, Kamego-Tome M, Zapata-Pachas MA, Alvarado GF. Association between body image dissatisfaction and depressive symptoms in adolescents. Braz J Psychiatry. 2017;39(4):316–22. doi: 10.1590/1516-4446-2016-1947 28355343 PMC7111410

[34] Ozmen D, Ozmen E, Ergin D, Cetinkaya AC, Sen N, Dundar PE, et al. The association of self-esteem, depression and body satisfaction with obesity among Turkish adolescents. BMC Public Health. 2007;7:80. doi: 10.1186/1471-2458-7-80 17506879 PMC1888702

[35] Almeida S, Severo M, Araújo J, Lopes C, Ramos E. Body image and depressive symptoms in 13-year-old adolescents. J Paediatr Child Health. 2012;48(10):E165-71. doi: 10.1111/j.1440-1754.2012.02576.x 22998142

[36] Divecha CA, Simon MA, Asaad AA, Tayyab H. Body image perceptions and body image dissatisfaction among medical students in Oman. Sultan Qaboos Univ Med J. 2022;22(2):218–24. doi: 10.18295/squmj.8.2021.121 35673292 PMC9155027

[37] Mohamed BAA, Idrees MHD. Body image dissatisfaction and its relation to body mass index among female medical students in Sudan: across-sectional study 2020-2021. BMC Womens Health. 2023;23(1):593. doi: 10.1186/s12905-023-02748-8 37950174 PMC10638698

[38] Medical student dies by suicide [Internet]. The Times of India; 2023 [cited 2025 Apr 30]. Available from: https://timesofindia.indiatimes.com/city/mangaluru/medical-student-dies-by-suicide/articleshow/105197522.cms

