# Supplementary material for: Mirror, mirror on the coast: Exploring body image perception and its nexus with self-esteem, mental well-being among student population at an education hub in South-India
Source: PLoS One. 2025 Jun 26;20(6):e0326171. doi: 10.1371/journal.pone.0326171 (PMC12200845; doi:10.1371/journal.pone.0326171)
Supplement: S1 File — (DOCX) [file pone.0326171.s001.docx]

**Perception of body image and its association with self-esteem, anxiety and depression among students**

1. **Socio-demographic variables**
2. Age (Years):
3. Sex: Male/female/others
4. Marital status: Unmarried/Married/prefer not to disclose
5. Religion: Hindu/Muslim/Christian/Others (specify)
6. Educational qualification:
7. Place of residence:
8. Type of family: Nuclear/ Joint/ 3 generation
9. Total family income per month (Approximate) (Rs):
10. Total no. of persons in the family:
11. **Anthropometry**

Height:_______ cm

Weight: _______ Kg

1. **Stunkard Figure Rating Scale**


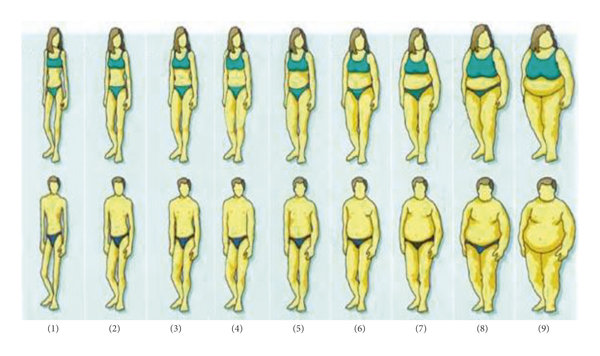


1. Identify the body image which you perceive to be having currently (from 1 to 9):
2. Indicate the body image which you wish to have (from 1 to 9):
3. **Subjective appearance evaluation (Sein Lee et al^a^ & Ehlinger PP et al^b^)**
4. What do you think your own body type is? 1 (Very skinny) 2(moderately skinny) 3(normal) 4(moderately fat) 5(very fat)
5. How attractive are you? On a scale of 1 to 4, of which 1 suggests very attractive and 4 suggests not at all attractive
6. **Body shape questionnaire**

1. **Rosenberg self-esteem scale**

1. **Hamilton-Anxiety rating scale**

1. Patient Health Questionnaire-9
